# Supplementary material for: Edge computing task scheduling mechanism based on multi-dimensional feature extraction and attention fusion
Source: PLoS One. 2026 Feb 24;21(2):e0343042. doi: 10.1371/journal.pone.0343042 (PMC12931809; doi:10.1371/journal.pone.0343042)
Supplement: Attachment — Submitted filename: Review_PONE-D-25-38912.pdf [file pone.0343042.s002.pdf]

Summary

This paper proposes a scheduling method for edge computing. It mixes graph-based modeling for how machines relate to each other with time-series models for how loads change over time. It aims to predict faults, keep tasks on time, and cut energy use. The authors compare against several baselines and report better accuracy, more completed tasks, and lower migration time.

What works well

1. The problem is practical and timely: fault-tolerant task scheduling at the edge.

2. Combining graph models with temporal models is sensible and well-motivated.

3. The paper outlines a clear high-level pipeline, which helps readers follow the flow.

Major issues to address

1. Data availability: PLOS ONE requires all underlying data to be public. The manuscript currently says data are available only on request. Please deposit the full dataset (or a faithful synthetic alternative that reproduces the results) in a public repository and include a DOI, plus all scripts used to produce the figures and tables.

2. Labeling and ground truth: Please explain exactly how faults/anomalies were labeled. Who labeled them, with what criteria, and at what sampling rate? Share the labeling protocol and code so others can reproduce it.

3. Baseline fairness and tuning: For each baseline, describe where the code came from (original repo vs. re-implementation), how you tuned hyperparameters, and whether all methods ran under the same conditions (hardware, time budget, number of trials). Consider a short appendix with the full baseline configs.

4. Statistics and reporting: For every table/figure, clarify what the "±" value means (SD or SE), how many runs you averaged over, and which random seeds you used. Please add confidence intervals and, where appropriate, statistical tests with a short justification.

5. Metric/units consistency: CPU utilization should be between 0–100%. Some tables appear to report values orders of magnitude larger or with unclear units. Please fix the units and double-check all reported numbers for consistency across the paper.

6. Cost model: The “cost (yuan)” and “unit task cost” metrics need a clear definition. State the pricing assumptions, time horizon, and how compute, migration, and failures contribute to cost. A short sensitivity analysis would help.

7. Thresholding logic: The text says a percentile multiplier raises thresholds to reduce false positives, but example values below 1 would lower them. Please reconcile the intent with the actual settings and justify the chosen percentile(s).

8. Graph formulation: You mention removing self-loops before GCN/GAT. Standard GCN usually adds self-loops so a node keeps its own features. Please justify removing them or adjust the formulation.

9. Loss design: Triplet/prototype loss seems to rely on labels derived from thresholder predictions, which can be circular. Please clarify whether you use ground-truth labels for the metric space and how you avoid confirmation bias.

10. Optimization details: List the exact optimizer(s), learning rates, schedulers, gradient clipping, batch sizes, training epochs, early-stopping criteria, and any weight decay used for all modules. If an unusually high learning rate is used anywhere, justify it.

Minor edits and quality improvements

1. Tighten the writing. Fix article usage, verb tenses, and a few awkward phrases. Reading the paper aloud once often catches these quickly.

2. Define all acronyms at first use. Keep a small glossary or table of symbols for readers outside the subfield.

3. Ensure all figures have clear axes, units, and legends that map each line to a metric. Consider increasing font sizes for readability.

4. Add an ablation study: (i) GCN only, (ii) GAT only, (iii) without self-attention, (iv) without the prototype/triplet loss. This will show which parts matter most.

5. Provide a short complexity and scalability note. Explain how training/inference scale with the number of hosts and graph density.
