# Supplementary material for: Edge computing task scheduling mechanism based on multi-dimensional feature extraction and attention fusion
Source: PLoS One. 2026 Feb 24;21(2):e0343042. doi: 10.1371/journal.pone.0343042 (PMC12931809; doi:10.1371/journal.pone.0343042)
Supplement: Attachment — Submitted filename: Point to point response (Reviewers).pdf [file pone.0343042.s004.pdf]

Major issues to address

1. Data availability: PLOS ONE requires all underlying data to be public. The manuscript currently says data are available only on request. Please deposit the full dataset (or a faithful synthetic alternative that reproduces the results) in a public repository and include a DOI, plus all scripts used to produce the figures and tables.

Reply:

We appreciate the reviewer's reminder to pay attention to PLOS ONE's data availability policy. We fully agree on the importance of data sharing for the research community. We have stored the complete dataset and corresponding scripts in the Dryad (https://doi.org/10.5061/dryad.wwpzgmszm).

2. Labeling and ground truth: Please explain exactly how faults/anomalies were labeled. Who labeled them, with what criteria, and at what sampling rate? Share the labeling protocol and code so others can reproduce it.

Reply:

We have added the following content in the "Experimental setup " section: Fault labels are generated based on a dynamic threshold function (see formula 2). When the utilization of any resource dimension (CPU, memory, disk) exceeds this threshold, it is marked as a fault state. The threshold is dynamically adjusted based on the 98% percentile of historical data, with an adjustment coefficient of 0.99999, to reduce false positives.

3. Baseline fairness and tuning: For each baseline, describe where the code came from (original repo vs. re-implementation), how you tuned hyperparameters, and whether all methods ran under the same conditions (hardware, time budget, number of trials). Consider a short appendix with the full baseline configs.

Reply:

We added in the 'Experimental setup’:

All baseline methods use the hyperparameters recommended in their original paper and run in the same hardware and software environment. All methods are run 100 times, and the average and standard deviation are taken. To ensure the fairness of experimental conditions and consistency of experimental results, each baseline method adopts a training framework similar to the results of this paper, with the main difference being the model architecture and anomaly detection strategy of each baseline method itself. All methods share the same data preprocessing process.

4. Statistics and reporting: For every table/figure, clarify what the “±” value means (SD or SE), how many runs you averaged over, and which random seeds you used. Please add confidence intervals and, where appropriate, statistical tests with a short justification.

Reply:

We have added relevant content in the 5.3 Comparative analysis of algorithms:

(9) Result analysis:

All algorithms were independently run 100 times in the same experimental environment to collect statistical data on key performance indicators. In algorithm comparative analysis, the value following "±" represents the half width of the 90% confidence interval. This reflects the fluctuation range of the indicator over multiple runs and is used to evaluate the stability of the results. Taking the confidence interval analysis of fault prediction, energy consumption, and task completion quantity as an example, the statistical information will be analyzed. In the comparative analysis of the fault prediction results in Table 4, the lower confidence interval (0.9262) of the F1 score (0.9328 ± 0.0066) of MFEAF is higher than the upper limit (0.9085) of the suboptimal algorithm DeepFT, indicating that the performance improvement is statistically significant. In the comparative analysis of energy consumption results in Table 5, the confidence interval for the average energy consumption of MFEAF (14.69 kWh) does not overlap with DeepFT (15.47 kWh) and TopoMAD (15.32 kWh), indicating a significant energy efficiency advantage. In the comparative analysis of task completion numbers in Table 6, the confidence intervals of MFEAF's total task count (131) and its interval task count (1.30 ± 0.19) are higher than other algorithms, indicating that its task processing ability is significantly better (with no overlapping intervals).

5. Metric/units consistency: CPU utilization should be between 0–100%. Some tables appear to report values orders of magnitude larger or with unclear units. Please fix the units and double-check all reported numbers for consistency across the paper.

Reply:

It needs to be modified, it's indeed incorrect. The CPU utilization units in Table 11 have been corrected to ensure that they are within the range of 0-100%.

(8) Average CPU utilization

The comparative analysis of average CPU utilization is shown in Table 11. MFEAF has the highest average CPU utilization rate (61.09%), leading other models by 3.63%~13.60%, indicating that its resource scheduling strategy can maximize the utilization of computing resources. ECLB performed the worst (47.49%), with a utilization rate 13.60% lower than MFEAF, and prominent resource idle issues, possibly due to uneven task allocation or excessive migration costs. TopoMAD and DeepFT have similar utilization rates (50.79% and 50.73%), which are about 10.3% lower than MFEAF. Overall, the high CPU utilization of MFEAF (61.09%) combined with its high task volume (131 tasks) (see Table 6) and low energy consumption (14.69 kWh) (see Table 5) indicates that it achieves a balance between performance and energy efficiency through efficient resource allocation.

6. Cost model: The "cost (yuan)" and "unit task cost" metrics need a clear definition. State the pricing assumptions, time horizon, and how compute, migration, and failures contribute to cost. A short sensitivity analysis would help.

Reply:

We will supplement the cost model definition in the 'experiment':

The container cost is calculated based on resource usage (CPU, memory, disk) and runtime, referring to the Alibaba Cloud container instance pricing model. The unit task cost is the total cost divided by the number of completed tasks.

7. Thresholding logic: The text says a percentile multiplier raises thresholds to reduce false positives, but example values below 1 would lower them. Please reconcile the intent with the actual settings and justify the chosen percentile(s).

Reply:

We have added relevant content in the "3.2 Data preprocessing module" section.

The adjustment coefficient is used to slightly lower the threshold to reduce false positives. Selection instructions for this value: (1) Numerical stability: When the data approaches the standardized upper limit, 0.99999 can slightly lower the threshold to avoid abnormal behavior caused by numerical accuracy issues. (2) The principle of minimum intervention: 0.99999 is very close to 1, and only minor adjustments are made to data that is close to saturation, without significantly changing the overall threshold distribution. (3) Balanced detection sensitivity: Combined with the subsequent 0.7 times threshold adjustment, a two-level threshold adjustment mechanism is formed, which ensures both numerical stability and sufficient anomaly detection sensitivity. Through this design, a good balance is achieved between ensuring numerical stability and detection performance.

8. Graph formulation: You mention removing self-loops before GCN/GAT. Standard GCN usually adds self-loops so a node keeps its own features. Please justify removing them or adjust the formulation.

Reply:

We have added relevant content in the " 3.3.1 Image data preprocessing module" section.

The removal of self-loops in this study is an application specific architectural decision aimed at better balancing the capture of relationships between nodes and the representation of node characteristics. Although standard GCNs typically add self-loops to preserve node self-information, in complex architectures such as MFEAF with multi-component fusion, residual connections and multi-layer networks can effectively capture and preserve node self-features. Meanwhile, it was found in the experiment that removing self-loops can improve the modeling ability of the model for inter node dependencies in edge task scheduling scenarios.

9. Loss design: Triplet/prototype loss seems to rely on labels derived from thresholder predictions, which can be circular. Please clarify whether you use ground-truth labels for the metric space and how you avoid confirmation bias.

Reply:

We have added relevant content in the " 4.1 Model Training Algorithm (2) Loss function " section.

The following discusses whether there is a cyclic dependency in triplet loss and how to avoid confirmation bias. The reason why this design does not have the problem of circular dependencies includes: abnormal labels are generated based on raw data and predefined thresholds before model training, not based on the predicted results of the model. The prototype vector will be updated based on sample features during the training process, but this is an iterative optimization process, not a cyclic dependency. Measures to avoid confirmation bias in this design include separating label generation from model training to ensure that labels are not affected by model predictions. By multiplying the threshold by 0.7, the detection sensitivity is increased to avoid confirmation bias caused by excessive reliance on strict thresholds. The prototype vector will be dynamically adjusted according to the training process, allowing the model to adapt to changes in data distribution rather than being fixed.

10. Optimization details: List the exact optimizer(s), learning rates, schedulers, gradient clipping, batch sizes, training epochs, early-stopping criteria, and any weight decay used for all modules. If an unusually high learning rate is used anywhere, justify it.

Reply:

We have added relevant content in the " 4.2 Scheduling optimization algorithm (4) Gradient optimization and scheduling projection (lines 11-17)" section.

Below is a detailed introduction to the AdamW optimizer, learning rate scheduler, and other components in the model. In the AdamW optimizer configuration, the model training optimizer lr=0.0005 is used to update the parameters of the deep learning model, including the weights and biases of the neural network. Decision optimization optimizer lr=0.8, used to optimize container scheduling decisions and find the optimal container allocation scheme, rather than training model parameters. In the configuration of the learning rate scheduler, the warm-up period is set to 10 epochs. The gradient clipping threshold is 1.0. The model learning rate is lr=0.0008, which is the default learning rate attribute defined in the model class and is mainly used for default settings during model initialization and loading. The weight decay value is 1e-4. When traversing the entire dataset, batch size is trained on a sample-by-sample basis.

This high learning rate is reasonable in decision optimization scenarios for the following reasons: (1) These high learning rates are not used for model weight training, but for optimizing scheduling decision variables. (2) Container scheduling is a discrete problem that requires rapid search for feasible solutions in a continuous optimization space. (3) Use cosine annealing learning rate scheduler to ensure a rapid decrease in learning rate from 0.8. Therefore, the high learning rate of 0.8 used in this study is specifically designed for container scheduling decision optimization tasks, combining cosine annealing scheduler and discretization steps to make it both effective and stable in this specific scenario. This setting is different from traditional neural network training and is a special strategy adopted to solve optimization problems in discrete decision spaces.

Minor edits and quality improvements

1. Tighten the writing. Fix article usage, verb tenses, and a few awkward phrases. Reading the paper aloud once often catches these quickly.

Reply:

We have read the entire text and revised the article, tense, and expression.

2. Define all acronyms at first use. Keep a small glossary or table of symbols for readers outside the subfield.

Reply:

We define all abbreviations when they first appear

3. Ensure all figures have clear axes, units, and legends that map each line to a metric. Consider increasing font sizes for readability.

Reply:

We ensure that all numbers have clear axes, units, and legends to improve readability.

4. Add an ablation study: (i) GCN only, (ii) GAT only, (iii) without self-attention, (iv) without the prototype/triplet loss. This will show which parts matter most.

Reply:

We have added relevant content in the " 5.2 Algorithm convergence analysis" section.

In the process of designing the network model, this study adopted a step-by-step design and validation strategy. The results showed that the complete MFEAF outperformed all variants in terms of performance indicators, indicating that each module contributed.

5. Provide a short complexity and scalability note. Explain how training/inference scale with the number of hosts and graph density.

Reply:

We have added relevant content in the " 5.3 Comparative analysis of algorithms (9) Result analysis" section.

The factors that affect the scalability of the algorithm in this article mainly include the number of tasks, the number of hosts, and the timing length. In terms of task scalability, the algorithm proposed in this paper exhibits a linear increase in task scalability. In terms of host scalability, complexity can be reduced through group attention or local perceptual fields. In terms of temporal length scalability, the results of this paper show a linear increase in task number scalability.
