# Supplementary material for: Edge computing task scheduling mechanism based on multi-dimensional feature extraction and attention fusion
Source: PLoS One. 2026 Feb 24;21(2):e0343042. doi: 10.1371/journal.pone.0343042 (PMC12931809; doi:10.1371/journal.pone.0343042)
Supplement: Attachment — Submitted filename: Point to point response.pdf [file pone.0343042.s005.pdf]

We thank the reviewers for their thoughtful comments and constructive suggestions, which have helped us improve the clarity and rigor of the manuscript. We have carefully addressed all points raised in the review.

-Very large percentage improvements looks exaggerated (6000%).Compared to MFEAF, DeepFT, TopoMAD, AWGG, PCFT, ECLB, and DFTM have improved by 103.6%, 1893.1%, 51.3%, 6000%, 734.9%, and 80.7%, respectively. Please check

Reply:

It has been modified as required. See (4) Average migration time.

The comparative analysis of migration time is shown in Table 7. MFEAF achieved the best performance in average migration time (0.14±0.03), with the lowest time consumption among all comparative methods, demonstrating its efficient and stable migration efficiency in edge dynamic environments. This advantage is attributed to MFEAF network model by integrating a graph attention network and a temporal network, which achieves accurate and efficient fault prediction and yields a better fault-tolerant scheduling optimization strategy, thereby significantly reducing the comprehensive cost of a single migration. This paper does not directly compare MFEAF with PCFT and TopoMAD in terms of migration efficiency, mainly due to the following considerations. PCFT is a virtual machine fault-tolerant coordination method for cloud computing environments, and its migration granularity and coordination mechanism are prone to causing a surge in migration times and durations in scenarios with limited edge resources and network fluctuations. TopoMAD relies on post-event fault detection results, and its passive recovery mode is prone to causing cascading migrations in dynamic edge environments, leading to a significant increase in migration costs.

Table 7 Comparative analysis of migration time

Algorithm	Total migration time	Average migration time
DeepFT	40.30	0.19±0.04
AWGG	29.95	0.20±0.07
ECLB	165.30	0.73±0.16
DFTM	35.77	0.29±0.07
**MFEAF**	**19.79**	**0.14±0.03**

-In the algorithm instead of see formula 23); may replace refer Eqn no

Reply:

It has been modified as required. See Table 1 and Table 2.

-May refer the following similar articles:

Reply:

The following literature has been cited in the introduction.

[16] Rathor V S, Singh M, Gupta R, et al. Towards Designing an Energy Efficient Accelerated Sparse Convolutional Neural Network[C]//2024 IEEE 36th International Conference on Tools with Artificial Intelligence (ICTAI). IEEE, 2024: 969-974.

[17] Behera S R, Panigrahi N, Bhoi S K, et al. Time series-based edge resource prediction and parallel optimal task allocation in mobile edge computing environment[J]. Processes, 2023, 11(4): 1017.
