# Supplementary material for: Edge computing task scheduling mechanism based on multi-dimensional feature extraction and attention fusion
Source: PLoS One. 2026 Feb 24;21(2):e0343042. doi: 10.1371/journal.pone.0343042 (PMC12931809; doi:10.1371/journal.pone.0343042)
Supplement: Attachment — Submitted filename: Point to point response-20260129 - 2.pdf [file pone.0343042.s006.pdf]

We thank the reviewers for their thoughtful comments and constructive suggestions, which have helped us improve the clarity and rigor of the manuscript. We have carefully addressed all points raised in the review.

- replace Eq (29)) with Eq. (29) and ensure consistent formatting for all equation references, particularly those used within the algorithm descriptions.

Reply:

The format for citing equations has been unified in Algorithm 1 and Algorithm 2.

- Table 4 does not appear in the paper. Please verify and correct this. Additionally, it is recommended to present Algorithms 1 and 2 explicitly as algorithm blocks, rather than referring to them as tables, to improve clarity

Reply:

(1) The order of all figures and tables has been checked and revised.

(2) The forms of Algorithm 1 and Algorithm 2 have been modified.

-The contributions of the paper are not clearly identifiable. It is essential to explicitly list the key contributions in bullet points to clearly highlight the novelty of the work.

Reply:

In the Introduction section, the contributions of this paper are described in three points.

The core contributions of this paper can be summarized as follows:

(1). A multi-dimensional feature extraction architecture that integrates graph attention networks with temporal networks is proposed. For the first time, the combination of graph attention networks and graph convolutional networks is achieved, and LSTM and self-attention mechanisms are introduced to jointly model the dynamic dependencies between edge nodes, thereby significantly

improving the accuracy of system state and fault prediction.

(2). Designed a training optimization mechanism incorporating adaptive learning rate and cosine annealing strategy. By dynamically adjusting the learning rate and incorporating the cosine annealing strategy, we effectively reduce the transmission of redundant information during training, accelerate model convergence, and enhance the adaptability and stability of the algorithm in dynamic edge environments.

(3). Realized end-to-end joint optimization of fault prediction and fault-tolerant scheduling. MFEAF not only achieves efficient fault prediction, but also outperforms existing benchmark methods in multiple dimensions such as energy efficiency, task completion, and migration cost through an integrated scheduling optimization algorithm.

Based on these innovations, MFEAF can effectively address issues such as low task execution success rates and high host energy consumption caused by limited device resources in edge computing environments, thereby enhancing the stability and resource utilization of edge computing systems.

-The conclusion section requires refinement to correct typographical and grammatical errors (e.g., phrases such as "improving traffic smoothness and safety; In the field of industrial Internet"). The section should be revised for clarity, and technical precision.

Reply:

The typographical and grammatical errors in the Conclusion section have been corrected.

To address the challenges of fault prediction and fault-tolerant scheduling caused by resource contention in resource-constrained edge computing networks, this paper proposes an edge computing task scheduling mechanism based on multi-dimensional feature extraction and attention fusion. MFEAF integrates Graph Attention Networks, Graph Convolutional Networks, Long Short-Term Memory networks, and Residual Networks to achieve accurate system-state prediction and dynamic dependency modeling. A model training algorithm is designed to optimize network parameters, and a scheduling optimization algorithm is developed to adjust scheduling decisions. Experimental results demonstrate that MFEAF significantly alleviates the problems of low task

completion rates and high host energy consumption caused by limited device resources in edge computing environments, thereby enhancing system stability and resource utilization.

-Please include a future scope or future research directions paragraph in the conclusion section, outlining potential extensions or improvements of the proposed work.

Reply:

A paragraph discussing future work has been added to the Conclusion section.

Although MFEAF demonstrates good performance in edge computing task scheduling, there are still several directions worth further exploration. Firstly, at the algorithm level, research can be conducted on lighter graph neural network structures to reduce computational overhead and adapt to edge devices with more limited resources. Secondly, in terms of application scenarios, MFEAF can be extended to areas with high requirements for real-time performance and reliability, such as intelligent transportation systems, industrial IoT, and smart healthcare, and customized optimization can be carried out in combination with domain knowledge. Finally, the integration with technologies such as federated learning and blockchain can be explored to achieve secure and trusted task scheduling and resource management in a distributed edge environment.

-The manuscript directly computes the Jain's Fairness Index without sufficient explanation. The role, significance, and relevance of this metric in evaluating fairness should be briefly discussed before presenting the calculation.

Reply:

An explanation of Jain's Fairness Index has been added before the Index analysis in Table 7.

To evaluate the fairness of scheduling algorithms in resource allocation, this paper adopts Jain's Fairness Index as the quantitative standard. Fairness specifically refers to the degree of balance in the instantaneous computing throughput (IPS, Instructions Per Second) obtained by active containers at each time step. IPS is mapped from CPU usage rates in real workload trajectories and

represents the actual computing power that containers can execute. The Jain Index measures whether the scheduler allocates limited computing resources reasonably and impartially among multiple concurrent containers. The calculation method of this index is given by formula (31). x_i represents the IPS value obtained by the i-th container at a specific time step, and n is the number of active containers. The value range of the Jain Index is $[\frac{1}{n},1]$, with a value closer to 1 indicating a more equitable allocation.

$$F(x_1,x_2,...,x_n)=\frac{(\sum_{i=1}^{n}x_i)^2}{n\cdot\sum_{i=1}^{n}x_i^2} \quad (31)$$

In the edge computing task scheduling scenario, the role of this metric is to transform abstract fairness into comparable numerical values through standardized calculation, objectively measuring the distribution balance of computing resources obtained by each host or container. The importance of this metric lies in its close correlation with system stability, energy efficiency, and quality of service (QoS): fair resource allocation can effectively prevent node overload, reduce the overhead of fault migration, and avoid task starvation, thereby enhancing overall system performance. In terms of relevance, this metric is particularly suitable for edge computing dynamic scheduling scenarios, as it can not only reflect the load balancing status in real time but also form a complete scheduling strategy evaluation system together with metrics such as task completion quantity, energy consumption, and migration time, providing a key basis for algorithm optimization and selection.

-Instead of using bracket-only citations such as Reference [12] or Reference [14] within the text, it is recommended to cite references using author names followed by “et al.” (e.g., Author et al. [12]) for improved readability.

Reply:

The description method of Reference citations has been modified as required in the Introduction section.
